# Developmental Perspectives on Arterial Fate Specification

**DOI:** 10.3389/fcell.2021.691335

**Published:** 2021-06-25

**Authors:** Dongying Chen, Martin A. Schwartz, Michael Simons

**Affiliations:** ^1^Yale Cardiovascular Research Center, Departments of Internal Medicine, Yale University School of Medicine, New Haven, CT, United States; ^2^Department of Cell Biology, Yale University School of Medicine, New Haven, CT, United States; ^3^Department of Biomedical Engineering, Yale University, New Haven, CT, United States

**Keywords:** arterial specification, vasculogenesis, angiogenesis, vascular remodeling, Notch activation, shear stress, VEGF signaling

## Abstract

Blood vessel acquisition of arterial or venous fate is an adaptive phenomenon in response to increasing blood circulation during vascular morphogenesis. The past two decades of effort in this field led to development of a widely accepted paradigm of molecular regulators centering on VEGF and Notch signaling. More recent findings focused on shear stress-induced cell cycle arrest as a prerequisite for arterial specification substantially modify this traditional understanding. This review aims to summarize key molecular mechanisms that work in concert to drive the acquisition of arterial fate in two distinct developmental settings of vascular morphogenesis: *de novo* vasculogenesis of the dorsal aorta and postnatal retinal angiogenesis. We will also discuss the questions and conceptual controversies that potentially point to novel directions of investigation and possible clinical relevance.

## 1. Introduction

**FIGURE 1:**
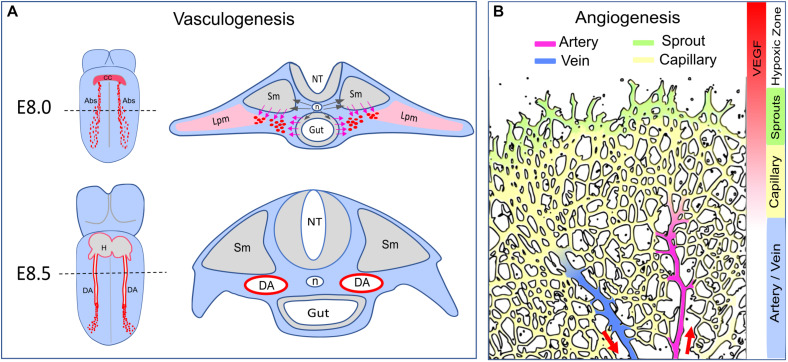
Arterial formation–vasculogenesis versus angiogenesis. **(A)** Formation of the dorsal aorta. Dash lines indicate transverse sections corresponding to the diagrams on the right. At E8.0, Shh (black arrows) released from the notochord (n) triggers VEGF expression in the somite (Sm) and the endoderm (Gut). The angioblasts (Abs, red dots) derived from the lateral plate mesoderm (Lpm) migrate toward the VEGF gradient (pink arrows) and coalesce as an endothelial cord. At E8.5, the dorsal aorta becomes a lumenized vessel connected to the heart (H). The developing vitaline veins and cardinal veins are omitted in this diagram. CC: cardiac crescent. NT: neural tube. **(B)** Arterial formation during postnatal angiogenesis in the retina. Vascular expansion is regulated by antiogenic sprouting at the vascular front, which is induced by a gradient of VEGF released from the neural bed in the avascular (hypoxic) zone. Artery formation is a process of vascular remodeling of the capillary bed. Red arrows indicate the direction of blood flow.

Acquisition of distinct arterial and venous identities is a critical step in vascular development. In the past two decades, numerous studies utilizing a variety of models, have placed Notch activation at the center of arterial specification. Studies exploring the role of Notch in arterio-venous (A/V) specification focused on three key areas: (1) how Notch is activated; (2) how Notch induces arterial fate; and (3) coordination of arterial fate acquisition with other developmental processes such as tubulogenesis and angiogenic sprouting. While VEGF-activation of Notch signaling has been considered the key step in arterial specification, recent findings centered on contributions of shear stress-induced Notch activation and cell cycle arrest to this process, raise questions about our traditional understanding of this subject. This review aims to integrate these new developments with the traditional A/V specification paradigm by carefully exploring studies of *de novo* vasculogenesis of the dorsal aorta (DA) during early embryogenesis and vasculature development in the neonatal mouse retina (Figures 1A,B). Since arterial specification in these two well-studied settings is subject to distinct regulatory mechanisms in distinct local microenvironments (Figures 1A,B), these two complementary models offer opportunities to address general vs specific features of this process. We will also examine key mechanisms responsible for Notch activation and their biological consequences. For a full discussion of signaling pathways involved in arterial and venous specification, readers can also refer to a number of excellent recent reviews (Lin et al., 2007; Roca and Adams, 2007; Aitsebaomo et al., 2008; Swift and Weinstein, 2009; Corada et al., 2014; Fish and Wythe, 2015; Simons and Eichmann, 2015; Mack and Iruela-Arispe, 2018; Red-Horse and Siekmann, 2019).

## 2. Acquisition of Arterial Fate During de novo Vasculogenesis– Formation of the Dorsal Aorta

During early embryogenesis, vasculature is established in the avascular embryo body. Nascent vessels are formed by emergence of undifferentiated endothelial cells (ECs) from mesodermal progenitors in a stepwise process defined as *de novo* vasculogenesis. Key steps in this process include (a) differentiation of mesodermal cells into angioblasts and ultimately into VEGFR2^+^/CD31^+^/VE-Cad^+^ ECs; (b) assembly of the newly emerged ECs into vascular cords; and (c) cord lumenization leading to establishment of blood flow (Risau and Flamme, 1995; Drake and Fleming, 2000; Crosby et al., 2005; Ferguson et al., 2005; Li et al., 2012; Fish and Wythe, 2015). In mouse embryos, extraembryonic progenitors of the splanchnopleuric mesoderm differentiate into blood islands and subsequently form the primitive vasculature in the yolk sac (Risau and Flamme, 1995; Ferguson et al., 2005; Ross and Boroviak, 2020). In the embryo body proper, the DA is formed by progenitors derived from the lateral plate mesoderm (Figure 1A). The DA is the first blood vessel (and the first artery) to form, by E8.25, at the 5 somite (5s) stage (Risau and Flamme, 1995; Drake and Fleming, 2000; Chong et al., 2011; Sato, 2013; Fish and Wythe, 2015; Figure 1A). Importantly, angioblasts begin expressing arterial markers Dll4 and Connexin 37 (Cx37) in the endothelial cord of the DA and subsequently gain more arterial markers such as Ephrin B2 and Connexin 40 during the lumenization process (Chong et al., 2011; Wythe et al., 2013; Herman et al., 2018). Slightly later, at approximately E8.5, other groups of mesodermal progenitors contribute to vasculogenesis of the endocardium, the sinus venosus, the vitelline veins and the cardinal veins, thus establishing a primordial circulatory system by the 13s stage (Chong et al., 2011; Fish and Wythe, 2015). At mid-gestation, the pharyngeal arch arteries, which form a rudimentary vascular apparatus that is ultimately remodeled into the aortic arch arteries, are derived from the second heart field mesoderm via vasculogenesis (Li et al., 2012; Wang et al., 2017; Kelly, 2021). Acquisition of arteriovenous identity, the next step during formation of these embryonic vasculatures, is subject to specific molecular controls in distinct and dynamically changing local microenvironments.

### 2.1 VEGF-MAPK Signaling Regulates the Initial Determination of Arterial Fate

Numerous studies in developing mouse, avians, xenopus and zebrafish embryos have revealed a mechanism involving non-cell autonomous signaling interactions among early embryonic tissues in close proximity, including the notochord, the hypochord (fish and xenopus only), the endoderm and the somites, to tightly control vasculogenesis of the DA (Hogan and Bautch, 2004). Briefly, Shh emanating from the notochord (mouse and zebrafish) and the endoderm (mouse and zebrafish) triggers VEGF expression from the endoderm (mouse and avian), the hypochord (xenopus), and/or the somites (mouse, xenopus, and zebrafish) (Hogan and Bautch, 2004; Sato, 2013; Fish and Wythe, 2015; Figure 1A). Once mesodermal precursors acquire the expression of VEGFR2 and differentiate into angioblasts, they migrate away from the lateral plate mesoderm toward the VEGF gradient and coalesce as an endothelial cord of the presumptive DA (Hogan and Bautch, 2004; Sato, 2013; Fish and Wythe, 2015; Figure 1A).

Both VEGFR2-null (Shalaby et al., 1995; Sakurai et al., 2005) and VEGFR2-Y1173F (Sakurai et al., 2005) mouse embryos die at E8.5 with no blood vessel having been formed, including the complete absence of the DA and the extraembryonic yolk sac vasculature, thus demonstrating the indispensable role of VEGF signaling at this time point. The role of Shh in the DA formation and Notch activation is believed to depend on VEGF (Hogan and Bautch, 2004; Castillo and Alvarez, 2011). Unlike VEGFR2-null embryos, the loss of Shh signaling in mouse embryos does not completely compromise DA formation as aggregates of angioblasts and a partially formed lumenized DA are present (Vokes et al., 2004; Coultas et al., 2010). In Shh-deficient embryos, Dll4 expression is reduced, particularly in the cranial portion of the DA, which corresponds to the loss of VEGF expression in the somites of the same segment of the embryo body (Coultas et al., 2010). Indeed, a defective DA formation in Shh-deficient embryos can be rescued by increasing VEGF signaling (Coultas et al., 2010). In zebrafish, Shh deficiency results in a complete loss of VEGF in the somites and Ephrin B2a expression in the DA, which can be rescued by artificial expression of VEGF (Lawson et al., 2002). Collectively, these observations suggest that Shh is required for VEGF expression and that that VEGF expression, in turn, determines Notch signaling and arterial identity of the DA.

The role of VEGF/VEGFR2 in arterial fate determination during DA formation is, however, more complex, as suggested by studies of VEGFR2 downstream signaling. Since phosphorylation of VEGFR2-Y1173 is the primary means of activation of ERK signaling, the lethality of VEGFR2-Y1173F mice has been interpreted to mean that ERK activation by VEGFR2 is indispensable for vasculogenesis. Yet careful analysis of data brings this into question. PLCγ1 is the key effector recruited by VEGFR2-Y1173 to transduce VEGFR2-MAPK signaling (Takahashi et al., 2001). PLCγ1-null mouse embryos exhibit embryonic lethality between E9.5–10.5 (Ji et al., 1997; Liao et al., 2002), 24–48 h after the formation of DA. Not surprisingly, the DA is formed in these PLCγ1-deficient mouse embryos (Ji et al., 1997). In zebrafish embryos with a y10 mutation (a loss-of-function mutation of PLCγ1), the DA forms, as visualized by vascular markers Fli1 and VEGFR2, but arterial markers EphrinB2 and Notch5 are low (Lawson et al., 2003). Ectopic expression of VEGF induces an increase in EphrinB2 expression in the wild type, but fails to do so in y10 fish (Lawson et al., 2003). Consistent with this study, characterization of other PLCγ1-deficient mutations in zebrafish also revealed defective arterial differentiation (Covassin et al., 2009). Given the role of PLCγ1 in transducing VEGFR2-Y1175 signaling to activate PKC-MAPK pathway, these studies support the conclusion that VEGF-MAPK signaling plays a principal role in acquisition of the arterial fate but is not essential for the initial DA formation.

VEGFR2 signaling activates both MAPK and PI3K-AKT pathways. It was proposed that VEGFR2 signals through MAPK to activate the arterial program, while the acquisition of venous fate involves PI3K-AKT activity (Hong et al., 2006; Lin et al., 2007; Fish and Wythe, 2015). Importantly, there is a cross-talk between Akt1 and MAPK cascades, with Akt-induced Ser259 phosphorylation of RAF1 inhibiting ERK activity (Ren et al., 2010). In HUVECs expressing RAF1-S259A, a mutation resistant to phosphorylation by AKT, ERK is constitutively activated, leading to the upregulation of the entire arterial program (Deng et al., 2013). Similarly, a suppression of PI3K-AKT signaling amplifies ERK activation and expression of arterial genes (Hong et al., 2006; Ren et al., 2010; Deng et al., 2013; Wythe et al., 2013). These findings suggest that the balance between PI3K/Akt and PKC/ERK is important for venous vs arterial identities.

There still is some uncertainty whether ERK is required for determination of the arterial fate during *de novo* vasculogenesis. In zebrafish embryos, biphosphorylated ERK (pERK1/2 and Thr202/Tyr204) is detected in the DA and the endothelial progenitors from the lateral plate mesoderm (Hong et al., 2006; Shin et al., 2016). Although experiments using chemical inhibitors suggested a functional role for ERK in arterial specification in zebrafish (Hong et al., 2006; Shin et al., 2016), interpretations of these data are confounded by the dosage and application time windows. ERK inhibition at an early embryonic stage (12 hpf) results in the absence of DA (Hong et al., 2006). In another study, ERK inhibitor at a lower dosage after 16 hpf downregulated Dll4 expression in the DA and the intersomitic angiogenic sprouts but did not affect Ephrin B2 expression (Shin et al., 2016). Besides the potential issue of specificity with chemical inhibition, this observation, however, does not provide a definitive answer to the question whether ERK is required for the initial acquisition of arterial fate. This is because ERK inhibition starts after the formation of DA, a time window when the blood flow can also possibly contribute (see section 2.2 below) and the initiation of ERK inhibition at earlier starting points (before 10 hpf) is not feasible due to severe developmental retardation and necrosis (Shin et al., 2016). A genetic model with a compound deletion of both ERK1/2 isoforms in VEGFR2+ angioblasts prior to the DA formation would be necessary to investigate the function of ERK signaling in regulation of arterial specification during this process.

### 2.2 Potential Role of Shear Stress in Post-vasculogenic Maintenance of Arterial Identity

The onset of blood flow during mouse embryogenesis begins at E8.25 (6–8s stage), shortly after lumenization of the DA (Ji et al., 2003; McGrath et al., 2003; Chong et al., 2011) as determined by the presence of a small number of red blood cells in the DA. This implies circulation since at E8.25 the yolk sac is the only hematopoietic organ capable of producing primitive erythrocyte (Ji et al., 2003; McGrath et al., 2003). Shear stress has been reported to be critical during embryonic vascular patterning. For example, defective hierarchical remodeling of the yolk sac vasculature is seen in Mcl1a null embryos with deficient contractility of cardiomyocytes (Lucitti et al., 2007). Titin-/- embryos with a weak and spontaneous heartbeat, also show defective lumenization of the DAs and the CVs (May et al., 2004; Gerull, 2015). These effects can be attributed to shear stress rather than oxygen transport because inhibiting blood cell formation, which reduces effective blood viscosity and thus shear stress, had similar effects, and can be rescued by injecting a dextran polymer to restore viscosity (Lucitti et al., 2007).

Does flow contribute to the regulation of arterial identity? It is reported that angioblasts express Dll4 and Cx37 prior to lumen formation in the DA in mouse embryos (Chong et al., 2011; Wythe et al., 2013; Herman et al., 2018). This suggests that arterial fate is predetermined in angioblasts when they aggregate into a solid endothelial cord that is as yet without apico-basal polarization and lumenization (Xu and Cleaver, 2011). After formation of a lumenized DA at E8.0–8.25 (4–8s), aortic ECs start to increase expression of arterial markers including Cx37/40, Dll4, Notch1/4, Hey1, and Nrp1 (Chong et al., 2011; Herman et al., 2018). Though lumenized at this stage, the DA is still a blind vessel as the vitelline vein and the cardinal vein are yet to be formed, meaning that systemic circulation has not been established (Chong et al., 2011; Sato, 2013). The initial acquisition of arterial fate thus occurs prior to the start of blood flow in the embryo proper, excluding shear stress as an important regulator. This conclusion is further supported by the observation that Cx40 and Dll4 are expressed in the endothelial cord of the DA at E8.25 in Rasip1-null embryos, which lack vascular lumens (Chong et al., 2011; Xu and Cleaver, 2011; Xu et al., 2011; Barry et al., 2016).

However, blood flow apparently contributes to the maintenance of arterial identity after embryonic blood circulation is established. Mouse embryos lacking the cardiac sodium-calcium ion exchanger Ncx1 do not have a heartbeat (and thus flow), leading to inhibition of EC Notch activation and expression of Cx40 and EphrinB2 in the DA (Hwa et al., 2017). Similarly, in the aforementioned Rapsi1-null embryos with compromised vascular lumen formation, arterial markers (potentially induced by VEGF) are expressed at E8.25 before flow would normally begin, but Cx40 expression is remarkably decreased at E9.0 when flow would be established in WT embryos (Chong et al., 2011). These observations clearly demonstrate that failure to establish effective blood circulation after 8s results in a decline of arterial identity in the DA, supporting a critical role for shear stress in maintenance of arterial identity.

In summary, VEGF determines the specification of artery fate during DA vasculogenesis, whereas shear stress maintains arterial identity after blood circulation is established. We suggest that VEGF expression from adjacent tissues (e.g., somites) is transient and declines after 8s, after which flow substitutes for VEGF to regulate arterial identity. This pattern resembles events in postnatal retinal angiogenesis, where VEGF initiates sprouting but after blood flow begins and VEGF levels drop, shear stress maintains vascular integrity (Roux et al., 2020).

## 3. Acquisition of Arterial Fate in Vascular Remodeling During Angiogenesis – Coordination of Arterial Specification and Sprouting

Unlike vasculogenesis, in which a bona fide endothelial lineage is established via mesodermal differentiation, angiogenesis refers to a distinct process of vascular patterning during which new vessels are formed by sprouting from pre-existing vessels (Gariano and Gardner, 2005; Stahl et al., 2010). In the mouse retina, morphogenesis of the retinal vasculature begins on postnatal day 0 (P0) from a tiny vessel at the optic nerve (Gariano and Gardner, 2005; Stahl et al., 2010). Its expansion during the first week after birth is driven by a gradient of hypoxia-induced VEGF released from the neural bed in the peripheral avascular zone (Gerhardt, 2008; Figure 1B). When a rudimentary capillary plexus is formed at P2, selected capillary ECs undergo arteriovenous differentiation to become the first arteries and veins (Gariano and Gardner, 2005; Stahl et al., 2010). Starting from P3, the retinal vasculature displays the following stereotypic structures: sprouts at the peripheral angiogenic front, a capillary bed back from the edge, and arteries and veins in an alternating arrangement (Gariano and Gardner, 2005; Stahl et al., 2010; Figure 1B). The radial expansion of retinal angiogenesis ceases at P7 when the superficial layer of vessels covers the entire neural bed and the peripheral avascular zone disappears (Gariano and Gardner, 2005; Stahl et al., 2010).

In contrast to the formation of the DA during which arterial fate is determined before the onset of flow, during retinal angiogenesis, arteries and veins emerge from a capillary plexus only in the presence of an effective blood circulation (Figure 1B). These results are, therefore, suggestive of the shear stress involvement. In these settings, arteriovenous differentiation transforms a non-hierarchical capillary plexus into a hierarchical vasculature, a process known as vascular remodeling. It is not clear what stimulus triggers the initial selection of ECs in the rudimentary plexus to undergo arteriovenous differentiation and form arteries and veins. Lineage tracing using inducible tip cell-specific Esm1-cre driver disclosed a trajectory of tip-capillary-artery movement (Xu et al., 2014; Pitulescu et al., 2017); by contrast, lineage tracing with artery-specific BMXcre showed that marked ECs remain in the arteries (Ehling et al., 2013). Thus, arterial ECs are derived from capillary ECs but not vice versa. The capillary-to-artery differentiation implies that tip ECs move against blood flow to enter the arteries again pointing toward a role for shear stress in A-V differentiation in this setting.

Similar principles likely apply to two additional instances of vascular development. The first was a follow up study to the work of Lucitti et al. (2007) that demonstrated a requirement for shear stress in remodeling of the primitive extraembryonic yolk sac vasculature in mouse embryos. Subsequent analysis revealed that vascular plexus ECs migrate against blood flow and coalesce to form major vessels as cardiac output (and, hence, shear stress) increases (Udan et al., 2013; Garcia and Larina, 2014). Likewise, in the embryonic pharyngeal arches at mid-gestation, the pharyngeal arch artery connected to the outflow tract of the heart is formed via vascular remodeling that transforms a non-hierarchical primordial vascular plexus into a major artery (Wang et al., 2017; Warkala et al., 2021), a process during which hemodynamic forces possibly play a role.

During angiogenesis, sprouting activity requires VEGF-regulated cell migration and proliferation (Gerhardt et al., 2003; Gerhardt, 2008; Okabe et al., 2014), while artery formation suppresses proliferation and angiogenic sprouting (Hasan et al., 2017). In other words, arterial specification does not occur at the vascular front where proliferation and sprouting are active. As both tip-stalk conversion and arterial specification are regulated by Notch signaling, the coordination of these two distinct biological processes in an angiogenic vasculature has remained a conundrum.

VEGF-induced Notch signaling regulates acquisition of the tip cell identity and tip-stalk conversion. At the angiogenic front where VEGF level is high, some ECs acquire tip cell identity through a competition mechanism (Ubezio et al., 2016). When an EC is activated by VEGF, it begins to express Dll4, leading to activation of Notch signaling in its neighboring cells. That, in turn, suppresses VEGFR2 expression and, thereby, responsiveness to VEGF, thus preventing these neighboring cells from becoming tip cells. This mechanism is in line with “lateral inhibition” observed in other developmental systems (Williams et al., 2006; Hellstrom et al., 2007b; Gerhardt, 2008; De Smet et al., 2009; Geudens and Gerhardt, 2011). This phenomenon can be recapitulated *in vitro* with an EC monolayer culture in which VEGF stimulation induces a “salt and pepper” pattern of heterogeneous Dll4 expression (Hellstrom et al., 2007a; Ubezio et al., 2016). Postnatal deletion of endothelial VEGFR2 results in the absence of tip cells and capillaries in the retinal vasculature, in which only major vessels are present (Zarkada et al., 2015; Pitulescu et al., 2017). Notch inhibition results in a hypersprouting phenotype that is blocked by VEGFR2-deletion (Zarkada et al., 2015), demonstrating that VEGFR2 signaling is required for angiogenic sprouting and is an essential upstream inducer of Notch signaling. While another study reported an opposite result (Pitulescu et al., 2017), it was likely due to incomplete deletion of VEGFR2. Interestingly, unlike the formation of DA during which VEGF-Notch determines arterial fate, in angiogenesis, sprouting ECs with high level of VEGF-induced Notch activation do not acquire arterial identity. This raises the yet unanswered question of how endothelial sprouts at the angiogenic front with high VEGF-Notch signaling avoid being ectopically arterialized?

## 4. Notch Signaling and Its Biological Consequences – Cell Cycle Arrest and Acquisition of Arterial Fate

**FIGURE 2:**
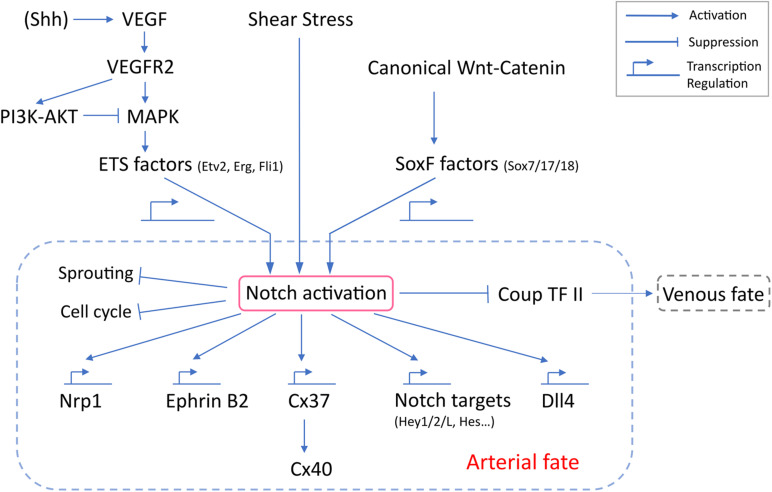
Molecular regulation of Notch activation and arterial specification. VEGF-MAPK activation of Notch depends on ETS factors, which transcriptionally regulate the expression of Notch genes. Canonical Wnt-catenin likely signals through Sox17 to activate Notch. SoxF factors regulates transcription of Notch genes. Despite reports implicating junctional mechanosensary proteins and the interaction with cytoskeleton in response to shear stress, how shear stress activates Notch remains poorly understood. Notch activation is a common downstream outcome and is the central determinant for arterial specification. Notch signaling induces a panel of arterial genes via transcription regulation. Notch also inhibits EC sprouting, induces cell cycle arrest and suppresses venous identity.

Notch signaling requires a direct cell-cell contact as both ligands (Dll1,3,4, Jag1, and 2) and receptors (Notch 1,2,3, and 4 in human and mouse, Notch1a, 1b, 2, and 3 in zebrafish) are transmembrane proteins (Kopan and Ilagan, 2009). Among these, Notch1 and 4 are specific to mouse arterial ECs (Reaume et al., 1992; Uyttendaele et al., 1996; Krebs et al., 2000), while Notch1a/b and 3 are expressed in zebrafish arteries (Lawson et al., 2001). Binding of a Notch ligand to its receptor triggers proteolytic cleavage by gamma-secretase and release of the Notch intracellular domain (NICD). Thus liberated, NICD translocates to the nucleus, where it interacts with RBPJ, Mastermind and other transcription regulators to transcriptionally control expression of target genes, including EphrinB2 (Grego-Bessa et al., 2007), Neuropilin1(Nrp1) (Sorensen et al., 2009), Cx37 (Fang et al., 2017), Dll4 (Caolo et al., 2010), Hey (Borggrefe and Oswald, 2009), and Hes (Borggrefe and Oswald, 2009) (Figure 2).

Notch activation regulates and is, in turn, regulated by Chicken Ovalbumin Upstream Promoter-transcription factor II (COUP-TFII), an orphan nuclear receptor critical for venous specification (Pereira et al., 1999; Polvani et al., 2019). A loss of endothelial COUP-TFII results in upregulation of Notch and arterial genes *in vivo* and in vitro (You et al., 2005; Chen et al., 2012). COUP-TFII overexpression suppresses Notch signaling and artery formation and promotes venous differentiation (Su et al., 2018). Artificial expression of Notch3 ICD inhibits COUP-TFII expression and prevents vessel differentiation to venous fate in zebrafish cardinal vein (Swift et al., 2014). These data point to the role of Notch activation in regulation of arterial vs. and venous fate specification.

Notch induces cell cycle arrest in multiple biological systems, including hair follicle cells (Deng et al., 2001; Shyu et al., 2009), T and B lymphocytes (Morimura et al., 2000; Joshi et al., 2009), keratinocytes (Rangarajan et al., 2001; Kolly et al., 2005), and cancer (Sriuranpong et al., 2001; Liao et al., 2018). Notch-induced cell cycle arrest in ECs was first implicated in the process of contact inhibition (Chen et al., 2000), during which higher EC density results in upregulation of Notch signaling and cell cycle arrest (Noseda et al., 2004). Artificial expression of Notch ligands Jag1 or Dll4, or the NICD in primary ECs from multiple vascular beds was found to induce cell cycle arrest at G0/G1 and regulate expression of cell cycle regulators (Noseda et al., 2004, 2005; Williams et al., 2006). Notch is also activated by arterial levels of laminar fluid shear stress (FSS), which contributes to the well-known suppression of EC proliferation in this setting (Fang et al., 2017). Further work has shown that cell cycle arrest in late G1 is critical for the arterial phenotype, whereas early G1 arrest is associated with venous specification (Chavkin et al., 2020).

What determines the effect of Notch in cell cycle arrest and arterial fate? A recent study (Fang et al., 2017) surprisingly identified Cx37 as the critical gene induced directly downstream of Notch ICD/RBPJ. Cx37 then induced the CDK inhibitor p27, possibly through a gap junctional transport-independent mechanism. The resultant cell cycle arrest was sufficient to allow arterial specification. This model was supported by a detailed epistatic analysis supporting the FSS-Notch-Cx37-p27 pathway in arterial fate specification.

A non-physiological, extremely high dosage of VEGF (1,000 ng/ml) has been reported to induce cultured EC monolayers to undergo synchronized oscillations in DLL4 expression and Notch activation, instead of the “salt and pepper” pattern seen with physiological VEGF levels (Ubezio et al., 2016). Similarly, intravitreal injection of high dosage of VEGF (300 ng) induced homogenous expression of Dll4 in the entire retinal vasculature at P5 Ubezio et al., 2016. Intriguingly, this treatment inhibited radial expansion and EdU labeling Ubezio et al., 2016, suggesting that synchronized Notch signaling serves as a negative feedback to suppress sprouting and proliferation. Conversely, Notch inhibition using neutralizing Dll4 antibody or Rbpj endothelial knockout reportedly enhanced ERK signaling (presumably induced by VEGF) in the hyperspouting vascular front (Pontes-Quero et al., 2019a; Pontes-Quero et al., 2019b). Surprisingly, high pERK signal correlated with increased expression of the cell cycle inhibitor p21 (Pontes-Quero et al., 2019a, b). Application of Dll4 neutralizing antibody induced proliferation in the capillary bed at 24 h but then strikingly suppressed by 48 h (Pontes-Quero et al., 2019a, b). This observation again suggests a negative feedback mechanism by which high VEGF-ERK signaling elicits a cell cycle arrest via p21, likely independently of Notch signaling.

## 5. Signaling Mechanisms That Activate Notch

Multiple studies have shown that Notch activation is regulated by expression of both its receptor and ligands. Loss of one or both alleles of Dll4 (Duarte et al., 2004; Gale et al., 2004; Krebs et al., 2004), Notch1 knockout (Swiatek et al., 1994), Notch1/4 double knockout (Krebs et al., 2000) or Rbpj knockout (Krebs et al., 2004; Nielsen et al., 2014) in the developing embryo decrease Notch signaling, leading to severe vascular defects. Conversely, increasing either Notch receptor or ligand expression or artificial expression of NICD (Corada et al., 2013) activates Notch signaling. Sequence analysis of putative regulatory regions of Dll4 and Notch4 genes shows binding motifs for ETS family transcription factors (e.g., Fli1 and ERG), RBPJ, β-Catenin, Sox17, and FoxC1/2, suggesting transcriptional regulation of Dll4 and Notch4 by these factors (Corada et al., 2010, 2013; Wythe et al., 2013; Fish et al., 2017). Upstream signaling pathways regulating these transcription factors, including Notch itself, potentially interact to regulate the transcription of Notch genes. In endothelial and non-endothelial systems, Notch signaling can positively regulate the expression of Notch ligands (Dll1, Dll4, and Jag1) and receptors (Notch 1 and 3), thus establishing a positive feed-forward loop (Weng et al., 2006; Qian et al., 2009; Caolo et al., 2010; Chen et al., 2010; Borggrefe and Liefke, 2012).

### 5.1. VEGF and ETS Family Transcription Factors

At the angiogenic front, VEGF-ERK-induced Dll4 expression and Notch activation are pivotal to regulation of tip-stalk conversion and provide an important negative feedback mechanism regulating VEGFR2 expression to prevent excessive sprouting (Williams et al., 2006). Notch inhibition by a gamma-secretase inhibitor DAPT results in hypersprouting and a hyperdense capillary network with increased ERK activation (Pontes-Quero et al., 2019a). This phenotype is a direct consequence of excessive VEGF-ERK signaling in the absence of Notch-mediated negative feedback. Moreover, macrophage-derived VEGF-C signaling through VEGFR3 has been reported to contribute additively to Notch signaling at the vascular front, as a loss of VEGFR3 leads to hypersprouting similar to Notch inhibition (Tammela et al., 2011).

Studies in HUVECs show that VEGF-ERK signaling can phosphorylate and activate ETS transcription factor ERG, which subsequently binds to DLL4 enhancers and initiates its transcription (Wythe et al., 2013; Fish et al., 2017). In particular, VEGF-activated ERK2 selectively phosphorylates ERG on serine 215 (Ser^215^), which peaks at 30 min and then declines, a kinetic pattern consistent with VEGF-induced phosphorylation of ERK (peaks at 5–10 min) and Dll4 expression (peaks at 1 h) (Fish et al., 2017). Activated ERG recruits transcriptional co-activator p300 to the Dll4 enhancer to regulate its expression (Fish et al., 2017). Given these observations *in vitro*, it was proposed that during vasculogenesis of the DA, VEGF-MAPK signaling induces Dll4 expression via ETS factor-regulated transcription (Wythe et al., 2013; Fish et al., 2017). However, ERG-deficient mouse embryos did not reveal strong phenotypes in DA formation and arterial specification. Erg exon4-null embryos die at E10.5–11.5 with vascular defects in multiple organs (Vijayaraj et al., 2012). But at E8.5, the DA is formed, despite a modest downregulation of Dll4 (Wythe et al., 2013). Another study reported that global knockout of Erg (Erg-null) is embryonic lethal only at E11.5–12.5, by which point the DA is normally formed (Fish et al., 2017). These observations suggest that vasculogenesis of the DA at E8.5 can be accomplished independent of Erg and that loss of Erg does not completely compromise angiogenic activity needed to support embryonic growth at E9.5–10.5. Postnatal EC deletion of Erg also results in mild reduction of vascular growth and branching in the retina (Fish et al., 2017; Shah et al., 2017). Nevertheless, in zebrafish, combined morpholino inhibition of Erg and Fli1a substantially downregulates Dll4 expression in the DA (Wythe et al., 2013), which suggests that the mild phenotype in Erg-deficient mice is likely due to compensation by other ETS factors.

Erg was found to regulate Notch signaling by balancing expression of Dll4 and Jag1 (Shah et al., 2017), two Notch ligands reported to play opposite roles in angiogenesis (Benedito et al., 2009). Both Dll4 and Jag1 genes have putative binding sites for Erg. Silencing ERG in HUVECs reduced Dll4 but upregulated Jag1 (Shah et al., 2017). The same phenotype is also found in the neonatal retina of mice with endothelial knockout of Erg, though to a lesser extent (Shah et al., 2017).

### 5.2. Shear Stress

Although studies of DA formation (see section 2.2) and retinal angiogenesis (see section 4) have suggested an important role for shear stress in Notch activation and arterial differentiation, how shear stress activates Notch remains poorly understood. Multiple studies have demonstrated that FSS increases cleavage of Notch 1 and 4, nuclear translocation of their ICDs and expression of target genes (Mack and Iruela-Arispe, 2018). Notch activation by FSS requires expression of its ligands on adjacent cells (Sweet et al., 2013; Polacheck et al., 2017), suggesting that FSS amplifies some aspect of this interaction. Consistent with its junctional localization, a number of studies suggest a connection to the junctional mechanosensory complex, consisting of PECAM-1, VE-cadherin and VEGFRs, that mediates an important subset of FSS responses (Tzima et al., 2005; Givens and Tzima, 2016; Tanaka et al., 2021). Chemical inhibition of VEGFR2 reportedly blocks flow activation of Notch (Masumura et al., 2009). Deletion of SHC, which is important for FSS signaling through the junctional complex also prevents flow activation of Notch (Sweet et al., 2013). The intermediate filament protein vimentin is also required for flow-induced Notch cleavage; this pathway reportedly involves phosphorylation of Vimentin and interaction with Jagged1 (van Engeland et al., 2019). Interestingly, vimentin is also required for signaling through the junctional complex via an interaction with PECAM-1 (Conway et al., 2013). These findings fit well with the close proximity of all these players in cell-cell contacts. Recent data posted on BioRXIV report a requirement for the adhesion GPCR latrophilin-2, another junctional component (Tanaka et al., 2020). Latrophilin-2 is a GPCR studied mainly in neurons (Moreno-Salinas et al., 2019) but also expressed in ECs (Camillo et al., 2020; Tanaka et al., 2020). This studied showed that it is required for flow activation of VEGFRs and downstream events via its GPCR function, and for flow activation of Notch signaling independent of G proteins (Tanaka et al., 2020). Importantly, latrophilin-2 is not required for EC responses to VEGF or Notch ligands in the absence of flow, indicating that it is critical for conferring flow sensitivity to these pathways (Tanaka et al., 2020). Despite these clues, a clear molecular mechanism is currently lacking.

In all of the above studies, Notch signaling contributes to endothelial stabilization, quiescence and arterial specification. In addition to the canonical function of the Notch ICD, junctional stabilization is mediated via a non-canonical mechanism in which the Notch1 transmembrane domain that remains after cleavage interacts with VE-cadherin to enhance barrier function (Polacheck et al., 2017). One limitation with current *in vivo* studies is that there is currently no specific way to inhibit flow activation of Notch signaling as opposed to complete inhibition via deletion of Notch ligands, receptors or other key players. The identification of latrophilin-2 as a component that specifically confers flow sensitivity to this pathway suggests an opportunity for approaches that do not globally inhibit. However, at present, a definitive study to demonstrate the *in vivo* function of flow-induced Notch activation is lacking.

### 5.3. SoxF Transcription Factors

The SoxF family transcription factors, Sox7, 17, and 18, are implicated in arterial specification. In zebrafish, Sox7 and Sox18 are expressed when lateral DAs fuse to form the single DA, while Sox17 is not detected at this stage (Cermenati et al., 2008). Though in Sox7-null fish embryos, the DA is formed with normal expression of Notch and arterial markers, loss of Sox7 leads to ectopic expression of VEGFR3, which is normally restricted to venous ECs (Hermkens et al., 2015). Also, an A-V shunt forms between the lateral DA and the cardinal vein, so that most of the flow bypasses the systemic blood circulation (Hermkens et al., 2015). The severity and penetrance of this phenotype is increased in Sox7; Sox18 double null fish embryos (Cermenati et al., 2008), suggesting functional redundancy in these two Sox F factors during early embryonic vascular patterning (Herpers et al., 2008).

In the mouse, Sox17 expression is enriched in developing and mature arteries and arterioles (Matsui et al., 2006; Corada et al., 2013; Zhou et al., 2015), while Sox7 and Sox18 are similarly expressed in arteries, veins and capillaries (Zhou et al., 2015). Similar to Sox7-deficient zebrafish, Sox17-endothelial null mouse embryos develop A-V shunts between the DA and CV (Corada et al., 2013). In the retina, postnatal deletion of Sox7, Sox17, or Sox18 in the endothelium leads to hyper-sprouting with dense capillaries with reduced arterial formation (Corada et al., 2013; Zhou et al., 2015; Kim et al., 2016). The similarity of this phenotype to Notch inhibition suggests possible effects of SoxF factors on Notch activation. Intriguingly, triple deletion of Sox7, Sox17, and Sox18 strikingly enhanced the hypersprouting phenotype with almost no artery formation (Kim et al., 2016), providing evidence of function redundancy among SoxF factors. Moreover, as a direct transcription factor targeting the non-coding regions of Dll4 and Notch4, Sox17 signals upstream of Notch (Corada et al., 2013). This conclusion is supported by the finding that inhibition of Notch *in vitro* and *in vivo* has little impact on Sox17 expression and that artificial expression of Sox17 in primary culture of venous ECs originally lacking Sox17 expression upregulates a panel of Notch and arterial genes (Corada et al., 2013).

It is not clear what upstream physiological stimulus activates the expression of Sox17 during arterial specification. Though VEGF signals upstream of Notch, it does not increase Sox17 expression (Corada et al., 2013). Interestingly, a recent study reported that non-physiological high dose-long duration VEGF treatment (50 ng/ml for 6–36 h) can strikingly upregulated Sox7 and Sox17 protein synthesis via the mTOR pathway without altering mRNA levels (Kim et al., 2016). Further investigations will be needed to understand the precise mechanism to regulate Sox F factors in arterial specification.

### 5.4. Canonical Wnt-Catenin Signaling

Upon binding of Wnt ligands to Frizzled receptors, β-catenin degradation is inhibited, its levels increase and β-catenin translocates into the nucleus where it functions as a transcriptional regulator (Dejana, 2010). Endothelial deletion of β-catenin is embryonic lethal at E12.5 (Cattelino et al., 2003), but at E9.5 when the DA is fully formed, embryos appear normal in size with no obvious cardiovascular abnormality (Wythe et al., 2013). Another study of β-catenin iECKO, however, reported mild hypersprouting at E9.5, similar to Notch inhibition (Corada et al., 2013). Though the Dll4 promoter region contains β-catenin binding sites, active canonical Wnt signaling was not detected in arterial ECs at E8.5 and E9.5 using Wnt reporter lines (Wythe et al., 2013), which explains the absence of or mild phenotype in this time window. These observations indicate that Wnt-catenin signaling is likely not required for the initial expression of Dll4 during vasculogenesis of the DA at E8.25 and instead functions mainly between E9.5–12.5.

Consistent with direct β-catenin binding to the promoter regions of Dll4 and Notch4 (Corada et al., 2010; Wythe et al., 2013), β-catenin is reported to activate Notch and induce arterial specification. In mouse embryos at E9.5 where β-catenin was genetically stabilized (GOF), sprouting was significantly reduced, with increased Notch signaling and ectopic arterialization (Corada et al., 2010). Additionally, A-V shunt formed between the DA and sinus venosus at E9.5 (Corada et al., 2010), further suggesting defects in A-V identity. Gain or loss of β-catenin signaling up or down regulated Sox17 expression and arterial markers in the postnatal retina and brain, consistent with a model in which Wnt-catenin induces Sox17, which induces Notch (Corada et al., 2013). Other than the endothelium, Wnt-catenin signaling also regulates Sox17 expression in endodermal development (Engert et al., 2013).

## 6. Conclusion

Arterial specification is a critical step in the process of vascular maturation during organogenesis and post-natal angiogenesis. Acquisition and maintenance of arterial identity in different organs in different developmental stages is subject to spatiotemporally distinct molecular mechanisms, with Notch activation being the common downstream determinant of arterial gene expression. Unlike arterial fate specification during organogenesis, arteriogenesis in postnatal setting is much less well understood. Though a wealth of studies has concluded that VEGF-ERK signaling determines arterial fate in angioblasts and in the nascent endothelial lineage during the formation of the DA, it remains uncertain whether the same mechanism applies during postnatal retinal angiogenesis or postnatal arteriogenesis in general. Comparing the DA vasculogenesis model with postnatal retinal angiogenesis is complicated by the concomitant development of angiogenic sprouting and vascular remodeling during the latter, in a microenvironment with a dynamically changing gradient of VEGF. Moreover, the complexity of retinal vasculature is amplified by the presence of shear stress that is also a critical inducer of Notch activation and arterial fate. Despite its obvious critical role, how FSS activates Notch and induces arterial fate specification is poorly understood. Future investigations will be necessary to provide a coordination in such complexity in order to fully understand this process.

## Author Contributions

DC wrote a complete draft with figures and legends. MS mentored the writing and revised the draft. MAS wrote section “Shear Stress” and edited the rest of the draft. All authors contributed to the article and approved the submitted version.

## Conflict of Interest

The authors declare that the research was conducted in the absence of any commercial or financial relationships that could be construed as a potential conflict of interest.
